# In Vitro–In Vivo Relationship in Mini-Scale—Enabling Formulations of Corallopyronin A

**DOI:** 10.3390/pharmaceutics14081657

**Published:** 2022-08-09

**Authors:** Tim Becker, Anna K. Krome, Sahel Vahdati, Andrea Schiefer, Kenneth Pfarr, Alexandra Ehrens, Tilman Aden, Miriam Grosse, Rolf Jansen, Silke Alt, Thomas Hesterkamp, Marc Stadler, Marc P. Hübner, Stefan Kehraus, Gabriele M. König, Achim Hoerauf, Karl G. Wagner

**Affiliations:** 1 Department of Pharmaceutical Technology and Biopharmaceutics, University of Bonn, Gerhard-Domagk-Str. 3, 53121 Bonn, Germany; 2 German Center for Infection Research (DZIF), Partner Site Bonn-Cologne, 53127 Bonn, Germany; 3 Institute for Medical Microbiology, Immunology and Parasitology, University Hospital Bonn, Venusberg–Campus 1, 53127 Bonn, Germany; 4 Department of Microbial Drugs, Helmholtz Centre for Infection Research, Inhoffenstr. 7, 38124 Braunschweig, Germany; 5 German Center for Infection Research (DZIF), Partner Site Hannover-Braunschweig, 38124 Braunschweig, Germany; 6 Translational Project Management Office (TPMO), German Center for Infection Research (DZIF), Inhoffenstr. 7, 38124 Braunschweig, Germany; 7 Institute for Pharmaceutical Biology, University of Bonn, Nußallee 6, 53115 Bonn, Germany

**Keywords:** absorption, amorphous solid dispersion, anti-infective, bioavailability, corallopyronin A, dissolution, in vitro drug testing, laboratory animals, pharmacokinetics

## Abstract

In vivo studies in mice provide a valuable model to test novel active pharmaceutical ingredients due to their low material need and the fact that mice are frequently used as a species for early efficacy models. However, preclinical in vitro evaluations of formulation principles in mice are still lacking. The development of novel in vitro and in silico models supported the preclinical formulation evaluation for the anti-infective corallopyronin A (CorA). To this end, CorA and solubility-enhanced amorphous solid dispersion formulations, comprising povidone or copovidone, were evaluated regarding biorelevant solubilities and dissolution in mouse-specific media. As an acidic compound, CorA and CorA-ASD formulations showed decreased solubilities in mice when compared with human-specific media. In biorelevant biphasic dissolution experiments CorA-povidone showed a three-fold higher fraction partitioned into the organic phase of the biphasic dissolution, when compared with CorA-copovidone. Bioavailabilities determined by pharmacokinetic studies in BALB/c mice correlated with the biphasic dissolution prediction and resulted in a Level C in vitro–in vivo correlation. In vitro cell experiments excluded intestinal efflux by P-glycoprotein or breast cancer resistance protein. By incorporating in vitro results into a physiologically based pharmacokinetic model, the plasma concentrations of CorA-ASD formulations were predicted and identified dissolution as the limiting factor for bioavailability.

## 1. Introduction

A formulation development which takes place early in the preclinical advancement of novel antibiotics is important to establish formulations which enable sufficient oral bioavailability (BA) to be administered during preclinical studies, clinical studies and beyond [1]. As it was for many other novel active pharmaceutical ingredients (API) during the early development phase, formulation development with limited amounts of API was critical to initiate the development of the antibiotic corallopyronin A (CorA) [2,3]. In particular, antibiotics often lack sufficient oral BA, resulting in low plasma levels and concentrations at the target site below the minimal inhibitory concentration that is required to deplete the respective pathogens. In 2019, 43% (175 projects) of novel antibiotics in the pipeline were, therefore, formulated for parenteral use and only 10% (41 projects) for oral use, despite the fact that parenteral administrations in the outpatient setting are linked with a higher effort for physicians and lower patient compliance [4,5,6]. To develop suitable formulations for the novel API CorA, a comprehensive analysis and understanding of the factors contributing to the absorption and BA of the novel API, alone and in different formulations, were, therefore, of utmost importance.

Prior investigations of the novel antibiotic CorA revealed an excellent activity in animal models of filariasis. This enabled treatment options against the neglected tropical diseases onchocerciasis and lymphatic filariasis, which are caused by the filarial nematodes *Onchocerca volvulus* (onchocerciasis) as well as *Wuchereria bancrofti*, *Brugia malayi* and *Brugia timori* (lymphatic filariasis). CorA inhibits bacterial DNA-dependent RNA polymerase and is therefore highly effective against Gram-positive and Gram-negative bacteria such as *Wolbachia*, endosymbionts of the pathogenic filariae [7,8,9,10,11,12,13]. Despite promising in vitro and in vivo results, CorA as a drug substance showed stability and solubility issues. CorA is highly permeable but has poor aqueous solubility at lower pH combined with a limited dissolution rate, based on its acidic character (pKa 3.7; logP 5.4; solubility at pH 1:0.11 µg/mL; solubility at pH 6.5:91.13 µg/mL; glass transition temperature: 5 °C) [14]. Embedding CorA in the polymers povidone or copovidone, resulting in amorphous solid dispersions (ASD) in the form of a glass solution, provided a stability- and solubility-enhanced formulation approach [14]. Thus, suitable formulation strategies for preclinical and clinical pharmacokinetic and pharmacodynamic studies were investigated.

The objective of the present study was to elucidate the BA of two CorA ASD-formulations in PK studies using BALB/c mice in comparison to the neat CorA. Inbred mice are often used for preclinical efficacy models due to their low material need, the availability of various knock-out strains or neutropenic models and their ease of handling [15,16]. Despite the pharmacodynamic relevance of these mice models, oral (PO) PK testing of solid oral formulation principles is challenging in such small organisms, i.e., limited sampling points and the applicable dosing volume of the formulation either as a solution or a suspension of the intended solid form. Therefore, in accordance with the 3R principle (replace, reduce, refine), in vitro and in silico methods were applied to build a sound physiologically based PK-model (PBPK) supporting future formulation optimization and potentially inter-species performance prediction [17,18,19,20]. However, a lack of appropriate experimental setups for mice does not allow correlation of in vitro methods and in vivo PK due to different pH profiles and physiologies of preclinical species and humans, resulting in variation in absorption and BA [20,21,22]. Thus, CorA and formulations thereof were investigated regarding species-specific biorelevant solubility and biorelevant dissolution combined with in silico PBPK modeling by implementing these in vitro experiments to forecast plasma concentrations and identify relevant factors for absorption and BA. In vitro cell experiments were performed to identify potential transporter interactions that could limit drug absorption in the intestine.

## 2. Materials and Methods

### 2.1. Chemicals and Materials

CorA was provided by the Helmholtz Centre for Infection Research (Braunschweig, Germany). Polyvinylpyrrolidone (Kollidon^®^ 30 LP) and vinylpyrrolidone-vinyl acetate copolymer (Kollidon^®^ VA 64) were kindly provided by BASF SE (Ludwigshafen, Germany). Ethanol 99.8% and tri-potassium citrate were purchased from Carl Roth GmbH & Co. KG (Karlsruhe, Germany). Tri-potassium phosphate, lecithin, 1-decanol and sodium taurocholate were purchased from Thermo Fisher GmbH (Kandel, Germany). Sodium oleate was purchased from Sigma (Darmstadt, Germany). Glycerol mono-oleate was kindly provided by Gattefosseé SAS (Saint-Priest, France). In addition, 1-micron full-flow polyethylene filters were purchased from Cole-Parmer GmbH. Acetic acid was purchased by Sigma Aldrich Chemie GmbH (Steinheim, Germany). Sodium hydroxide was purchased from VWR International, LLC (Darmstadt, Germany). Water for injection was purchased from Fresenius Kabi Deutschland GmbH (Bad Homburg, Germany). LC-MS grade acetonitrile and water were purchased from Bernd Kraft GmbH (Duisburg, Germany), and LC-MS grade ammonium acetate from Merck KGaA (Darmstadt, Germany). Cell culture reagents were purchased from Sigma Aldrich (Taufkirchen, Germany).

### 2.2. Manufacture of the Spray-Dried CorA-Amorphous Solid Dispersion Formulations

To obtain ASDs, CorA and the polymers povidone or copovidone were dissolved in ethanol targeting a solid load of 10% (*w*/*v*) with a corresponding drug load of 20%. The spray-dried ASDs were prepared using a B-290 mini spray dryer connected to an Inert Loop B-295 and a dehumidifier B-296 (BÜCHI, Flawil, Switzerland). Nitrogen was used as an inert drying gas. The parameters were set at: feed rate of solution 5 mL/min; inlet temperature 85 °C; outlet temperature 59 °C; nitrogen spray gas flow 30 mm (corresponding to 357 L/h) and aspirator rate set to 100% corresponding to 35 m^3^/h. Yields of the spray-drying process were 60–65%. The spray-dried powder was dried afterwards for 48 h at 40 °C under vacuum using a vacuum oven (Binder GmbH, Tuttlingen, Germany) to remove residual solvent.

### 2.3. Solubility Determination in Biorelevant Medium

The kinetic solubility of neat CorA, CorA-povidone-ASD and CorA-copovidone-ASD were determined by using the shake flask method in two different setups, monitoring CorA concentrations between 2 min and up to 4 h. FaSSIF-V2- and FeSSIF-V2-media were used based on species-specific pH values. For setup 1, the established pH values for FaSSIF-V2 (pH 6.5) and FeSSIF-V2 (pH 5.8) were used to represent the pH values in the human gut [23]. Setup 2 corresponded to the mouse gut pH, resulting in FaSSIF-V2-mouse (pH 5.2) and FeSSIF-V2-mouse (pH 4.8) media [24]. For both approaches, an excess of neat CorA or CorA formulation was introduced into 10 mL of the respective biorelevant media (Table 1) and incubated for 4 h (setup 1) or 1 h (setup 2) in a GFL 1083 shaking incubator (Gesellschaft für Labortechnik GmbH, Burgwedel, Germany) at 37 °C. The endpoints after 4 h (setup 1) and 1 h (setup 2) represent the intestinal transit times of humans (4 h) and mice (1 h) [25,26]. Samples (0.5 mL) were withdrawn after 2 min and the respective endpoints and centrifuged for 5 min at 21,000 g and 37 °C. The supernatant was diluted 10-fold with methanol to avoid precipitation and quantified by high-performance liquid chromatography (HPLC) (Section 2.6). Kinetic solubility profiles were established in triplicates and the statistical significance on a difference in solubilities was assessed using a one-way analysis of variance (ANOVA; data were analyzed using GraphPad Prism, version 8.0, San Diego, CA, USA) with a significance level of α = 0.05.

### 2.4. Biorelevant Dissolution

#### 2.4.1. Monophasic Dissolution

Non-sink dissolution of CorA-ASD formulations were performed under biorelevant mouse conditions. The water bath was kept at 37 ± 0.5 °C. In order to mimic biorelevant conditions, the experimental design was adapted to the in vivo-used species mouse concerning the pH profile and transit time [24,27]. Moreover, the fed state was assumed to be the most appropriate condition due to the free food access for the mice during PK studies. Since no appropriate test media mimicking the gastrointestinal conditions of mice are described in literature, human FeSSIF-V2 was adjusted to the reported mouse pH in a fed state similar to the procedure applied by the GastroPlus PBPK software [23,28]. The resulting composition of the FeSSIF-V2-mouse is depicted in Table 2. To mimic dissolution in the stomach, 50 mL of aqueous dissolution media was set to pH 3.0 using a buffer concentrate based on McIlvaine buffer (Table S1) [29]. A sample size of 10 mg neat CorA and 50 mg of CorA-ASD formulation (drug load 20%), respectively, were tested. After 20 min, the approximate stomach transit time in mice, FeSSIF-V2-like concentrate was added to shift the pH from pH 3.0 to 4.8, simulating the small intestine pH of mice [30]. Dissolution testing was performed for 80 min based on the stomach and small intestine transit time in mice [26]. The concentration was monitored online with an Agilent 8454 UV-Vis spectrophotometer (Waldbronn, Germany), using the first derivative at 394 nm in order to eliminate scattering artefacts. Due to possible undissolved or precipitated particles, the aqueous phase was filtered using a 1-micron full-flow filter. Three independent dissolution tests were performed for each sample.

#### 2.4.2. Biphasic Dissolution

**Figure 1:**
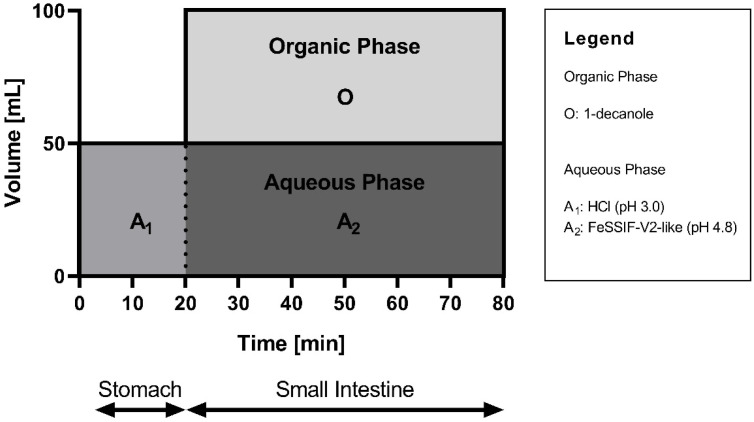
Biphasic dissolution setup for the mouse model.

The dissolution experiment of neat CorA and CorA-ASD formulations were performed using the biphasic dissolution apparatus BiPHa+ [31,32]. The experimental setup for the aqueous phase was identical to the monophasic setup (Section 2.4.1). Additionally, 50 mL of 1-decanol were added simultaneously to the pH shift after 20 min from pH 3.0 to pH 4.8 covering the aqueous phase [23]. The concentration profiles of both phases were measured online continuously, with an Agilent 8454 UV-Vis spectrophotometer, similar to Section 2.4.1. Figure 1 shows the experimental setup of the biphasic dissolution.

### 2.5. Pharmacokinetic Study Setup

The animal experiment was conducted according to European Union Directive 2010/63/EU and was approved by the State Agency for Nature, Environment, and Consumer Protection North Rhine-Westphalia, Germany, (AZ 84-02.04.2015.A507). Female BALB/c mice (6–8 weeks old) were obtained from Janvier (Le Genest-Saint-Isle, France). The animals were housed at the animal facility of the Institute for Medical Microbiology, Immunology and Parasitology at the University Hospital Bonn, Germany. The mice had free access to food and water over the course of the entire experiment. Neat CorA and the solid CorA-povidone- and CorA-copovidone-ASD formulations were suspended in water immediately prior to administration (CorA 36 mg/kg, volume 10 mL/kg). The dose for the pharmacokinetic study was based on efficacy studies in BALB/c mice. Since 36 mg/kg was able to deplete the Wolbachia more than 95%, the dose was kept constant. An oral gavage was used for all oral administrations. Blood samples from the tail tip were collected after 5, 10, 15, 30, 60, 180 and 480 min. Section 2.6 describes the processing of the samples and bioanalysis of the blood samples.

### 2.6. Bioanalysis of Corallopyronin A

The collected blood samples were centrifuged for 10 min at 4 °C and 3220 g. The generated plasma was removed and mixed in a ratio of 1:3 with ice-cold acetonitrile. The mixture was vortexed for 10 s and centrifuged for 25 min at 4 °C and 11,600 g. The plasma concentrations were quantified by HPLC using an Alliance e2695 separation module and the 2998 PDA detector (Waters, Eschborn, Germany). A Waters XBridge^®^ Shield RP18 column (3.5 µm, 2.1 × 100 mm, 130 A) was used at 30 °C. Data were analyzed by Empower 3 software and quantified using an external reference standard. A solvent gradient was used comprising mobile phase A (acetonitrile/water 5/95 with 5 mM ammonium acetate and 40 µL acetic acid per liter) and mobile phase B (acetonitrile/water 95/5 with 5 mM ammonium acetate and 40 µL acetic acid per liter) with a gradient from 70%A/30%B to 20%A/80%B, stepwise within 30 min and a flow rate of 0.3 mL/min. A sample volume of 5 µL was injected and quantified at a wavelength of 300 nm.

### 2.7. Pharmacokinetic Analysis

The extrapolated area under the plasma concentration curve (AUC_0–inf_) was calculated using the PKPlus™ Version 9.7/2.5 (Simulations Plus, Inc., Lancaster, CA, USA) applying a non-compartmental approach. The maximal plasma concentration (C_max_) and the associated time (T_max_) were directly determined from the corresponding plasma concentration time profiles. An intravenous (IV) pharmacokinetic profile including pharmacokinetic (PK) parameters was already reported by Krome et al. [14]. For the absolute BA calculation of the oral formulations Equation (1) was used. All values are presented in median (±IQR).

BA (%) = abs. Bioavailability

(1) $$\mathrm{BA}\;\left(\%\right)=\frac{\mathrm{AU}C_{0–\inf}\;\mathrm{PO}}{\mathrm{AU}C_{0–\inf}\;\mathrm{IV}}\cdot 100$$

### 2.8. Cell Culture Experiments

For the in vitro investigation of the interaction of CorA with P-glycoprotein (P-gp) and the breast cancer resistance protein (BCRP), three different cell lines were used: MDCK II parental and the transfected cell lines which overexpress P-gp or BCRP: MDCK II MDR1 and MDCK II BCRP, respectively. The cells were a generous gift from Dr. A. Schinkel (The Netherlands Cancer Institute, Amsterdam, The Netherlands). The cells were cultured and kept in Dulbecco’s Modified Eagle’s Medium (DMEM) enriched with 10% fetal bovine serum, 2 nM L-glutamine, 50 µg/mL streptomycin and 50 U/mL penicillin G. The cells were stored in a humidified incubator at 37 °C containing 5% CO_2_. Sub-culturing was performed after the cells reached a confluence of approximately 90%. The cells were detached by the addition of 0.05% Trypsin and 0.02% EDTA. After centrifugation (4 min at 266 g and 4 °C) the supernatant was removed and the cell pellet was re-suspended in fresh medium. For the cell culture experiments, the cells were washed twice with phosphate-buffered saline (PBS) and then counted using a CASY1 model TT equipped with a 150 µm capillary (Schaerfe System GmbH, Reutlingen, Germany).

#### 2.8.1. Investigation of Active Transport via P-gp and BCRP

The amount of accumulated CorA in the single cells was determined for the MDCK II parental, MDCK II MDR1 and MDCK II BCRP cells. The cells were prepared as described above and suspended at a final cell density of 250,000 cells per mL together with CorA at different concentrations (0.25, 0.5, 0.75 and 1 µM) and incubated for 1 h (37 °C and 5% CO_2_). After reaching the intra- and extracellular concentration equilibrium, single cell fluorescence was measured on the Red/V channel using a Guava easyCyte 8HT flow cytometer. Potential efflux of CorA due to its interaction with P-GP or BCRP was identified by different fluorescence intensities of parental and overexpressing cells due to different amounts of accumulated CorA. Conversely, similar fluorescence intensities indicated no interaction.

#### 2.8.2. Investigation of the Interaction with Hoechst 33342

Hoechst 33342 is a fluorescent substrate of P-gp which has a significant increase in fluorescent intensity when bound to DNA or embedded in a lipophilic environment like the cell membrane [33]. The effect of CorA on the transport of Hoechst 33342 was investigated using MDCK II MDR1 cells. Different CorA concentrations (3.16 and 10 µM) were given together with varying concentrations of Hoechst 33342 (1, 1.5 and 2 µM). As a control, the transport of Hoechst 33342 in the absence of CorA was included. The fluorescence was measured with a POLARstar microplate reader (BMG Labtech, Offenburg, Germany) in constant 1 min intervals up to 60 min, at an excitation/emission of 355/460 nm. The association kinetic of interaction between Hoechst 33342 and P-gp with and without CorA was analyzed using a one-phase association fit (data were analyzed using GraphPad Prism, version 8.0, San Diego, CA, USA).

### 2.9. PBPK Modeling

Plasma concentration-time profiles for CorA-povidone and CorA-copovidone ASD formulations were simulated using the PBPK modeling software GastroPlus™ 9.8 (Simulations Plus, Inc., 2020). Data describing the physicochemical properties and the physiology of the investigated species were obtained from in vitro studies and from estimates calculated by ADMET predictor 9.5 (Simulations Plus, Inc., 2019). Physicochemical properties of CorA, including molecular weight, pKa, logP and pH dependent solubility were recently published [14]. Additional biopharmaceutic relevant properties such as biorelevant solubility, dissolution and P-gp or BCRP interaction are described in this work. Shake flask solubilities after 1 h (Section 2.3) were used as the input for biorelevant solubilities to consider solubilities after the small intestine transit. First simulations were based on an immediate-release suspension as the dosage form. In the following, dissolution results (Section 2.4.1) were incorporated to the model via Weibull parameters calculated from the monophasic dissolution setup. The percentage of released dose is described in Equation (2) [34].

(2) $$\%\mathrm{Dose}\;\mathrm{Released}=\;\mathrm{Max}\left(1-e^{\left\{1-\frac{-\left(t-T\right)b}{A}\right\}}\right)\;$$

where Max = maximum of released API, T = time lag, b = shape factor of the curve and A = time scale. For PBPK modelling, the observed PK data of oral administration of ASD formulations administered as an aqueous suspension were used from PK studies in mice. The IV data provided the elimination and distribution behavior of CorA. Fed state was assumed due to the free food access of the mice. A detailed summary of the input parameters is provided in the Supplementary Materials (Table S2). Prediction errors (PE) for the PK parameter C_max_ an AUC_0–inf_ were calculated using Equation (3) [35].

(3) $$\%\mathrm{PE}=\frac{\mathrm{observed}\;\mathrm{value}-\mathrm{predicted}\;\mathrm{value}}{\mathrm{observed}\;\mathrm{value}}\cdot 100$$

## 3. Results

### 3.1. Solubility Determination in Biorelevant Medium

The solubilities of CorA and CorA-ASD formulations in the biorelevant media FaSSIF-V2, FeSSIF-V2, FaSSIF-V2-mouse and FeSSIF-V2-mouse media were measured (Table 3). Sampling points were chosen to determine the initial solubility after 2 min in comparison with the solubility after the small intestinal transit (human: 4 h; mouse: 1 h). In both media representing the human gut conditions (Table 3), the initial solubilities of the CorA-ASD formulations (CorA-povidone: 1.09/1.06 mg/mL; CorA-copovidone: 0.96/0.67 mg/mL) were significantly higher (CorA-povidone: *p* < 0.0001; CorA-copovidone: *p* < 0.0001) than those of neat CorA (0.00/0.00 mg/mL). No significant difference was detected between the ASD formulations in FaSSIF-V2 (*p* = 0.8876). However, in case of the simulated fed state, CorA-povidone had a significantly higher initial solubility after 2 min (*p* < 0.0001). Moreover, the ASD formulations achieved high solubility values of CorA-povidone: 1.24/0.94 mg/mL and CorA-copovidone: 1.40/0.93 mg/mL for at least 4 h.

The biorelevant solubilities for CorA-ASD formulations were also measured in FaSSIF-V2-mouse and FeSSIF-V2-mouse media (Table 3). While for both media the solubility enhancement of the ASD-formulations was demonstrated (CorA-povidone: *p* < 0.0001; CorA-copovidone: *p* < 0.0001) the solubility in FaSSIF-V2-mouse media showed lower values after 2 min (CorA-povidone: 0.11 mg/mL; CorA-copovidone: 0.09 mg/mL) when compared with the FeSSIF-V2-mouse (CorA-povidone: 0.66 mg/mL; CorA-copovidone: 0.45 mg/mL). No dissolved CorA was detected for neat CorA. In FaSSIF-V2-mouse media, a lower concentration for CorA-povidone (0.07 mg/mL) was observed after 1 h, whereas for CorA-copovidone the solubility further increased (0.19 mg/mL). In the FeSSIF-V2-mouse no significant differences were detected after 1 h for either formulation (*p* = 0.3080), indicating stable supersaturation. For neat CorA, the concentrations after 2 and 1 h were ≤0.02 mg/mL. Solubilities after 1 h were used as input parameters for the PBPK modelling of the CorA-ASD formulations.

### 3.2. Biorelevant Dissolution

#### 3.2.1. Monophasic Dissolution

**Figure 2:**
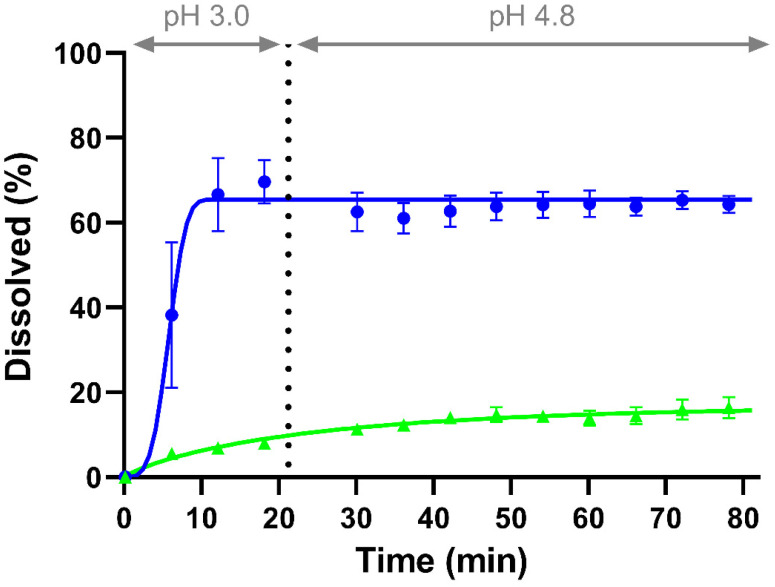
Monophasic dissolution experiment of CorA-povidone (●) and CorA-copovidone (▲) under biorelevant conditions. Lines represent the Weibull fit.

Dissolution profiles of CorA-ASD formulations with a Weibull function fit were generated (Figure 2). CorA-povidone showed an initial dissolution of 70% at pH 3.0 meeting an equilibrium at 65% until the end of dissolution (Table 4). At pH 3.0 CorA-copovidone achieved 8% of dissolved drug that increased to 16% by the end of the dissolution.

#### 3.2.2. Biphasic Dissolution

**Figure 3:**
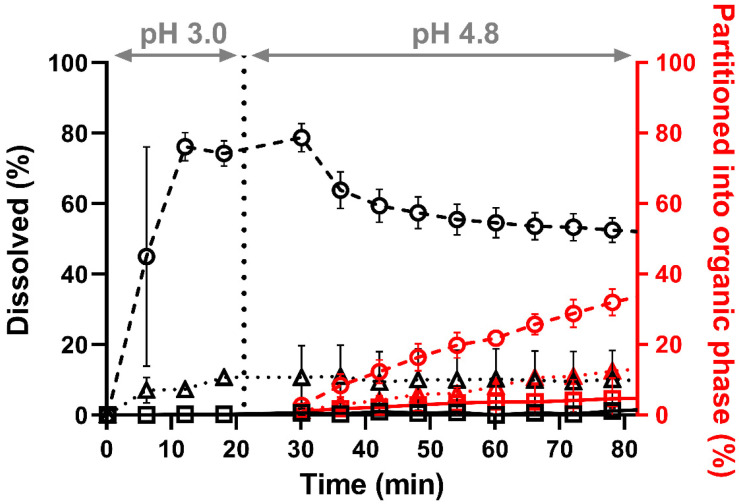
Biphasic dissolution experiment under mouse gut conditions; neat CorA (□; solid line), CorA-povidone (○; dashed line) and CorA-copovidone (△; dotted line). *n* = 3 (mean ± SD).

The dissolution performances of neat CorA, CorA-povidone-ASD and CorA-copovidone-ASD were investigated using the biphasic dissolution tool BiPHa+ combined with biorelevant media (FeSSIF-V2-mouse) and a species-specific pH shift. The dissolution profiles of the aqueous and the organic phases were determined (Figure 3). For neat CorA < 1% was dissolved in the aqueous phase during the first 20 min at pH 3.0, while after the pH shift to pH 4.8 a maximum of 2% after 80 min was observed. Accordingly, partitioning into the organic phase was limited due to the low amount of CorA dissolved in the aqueous phase, reaching only 4% partitioning after 80 min. By using the ASD formulation approach, in vitro biphasic dissolution exhibited enhanced dissolution performances. However, differences between the formulations were observed in the stomach part at pH 3.0. In contrast to the monophasic dissolution after the pH shift from pH 3.0 to pH 4.8, the aqueous phase was covered with the organic phase, resulting in a distribution sink, mimicking in vivo absorption. The aqueous concentration for the CorA-povidone dissolution increased to 78% followed by a decrease to approx. 55% within 30 min. In comparison, the aqueous concentration of the CorA-copovidone dissolution resulted in 10% by the end of the experiment. As only dissolved API in the aqueous phase was available for partitioning into the organic phase, CorA-povidone reached 34% of partitioned CorA after 80 min, whereas for CorA-copovidone only 13% was observed.

### 3.3. Pharmacokinetic Study

**Figure 4:**
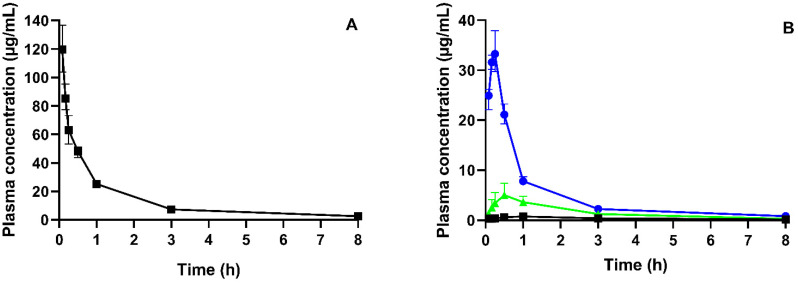
CorA plasma concentration time profiles in BALB/c mice: (**A**) IV administration of a CorA-solution (36 mg/kg) (Data from Ref. [14]); (**B**) PO administration of neat CorA (■), CorA-povidone (●) and CorA-copovidone (▲) (36 mg/kg), administered as an aqueous suspension in female BALB/c mice (median ± IQR, *n* = 4 per group).

Plasma profiles after IV-administered neat CorA and PO-administered neat CorA and CorA-ASD formulations in BALB/c mice (*n* = 4 per group) and associated PK parameters were generated (Figure 4 and Table 5, respectively). The IV administration allowed the calculation of the abs. BA (Equation (1)) and yielded 3% for neat CorA, 33% for CorA-povidone and 10% for CorA-copovidone. A later T_max_ was determined for neat CorA (1 h) compared to the ASD formulations (10 min for CorA-povidone and 30 min for CorA-copovidone).

**Figure 5:**
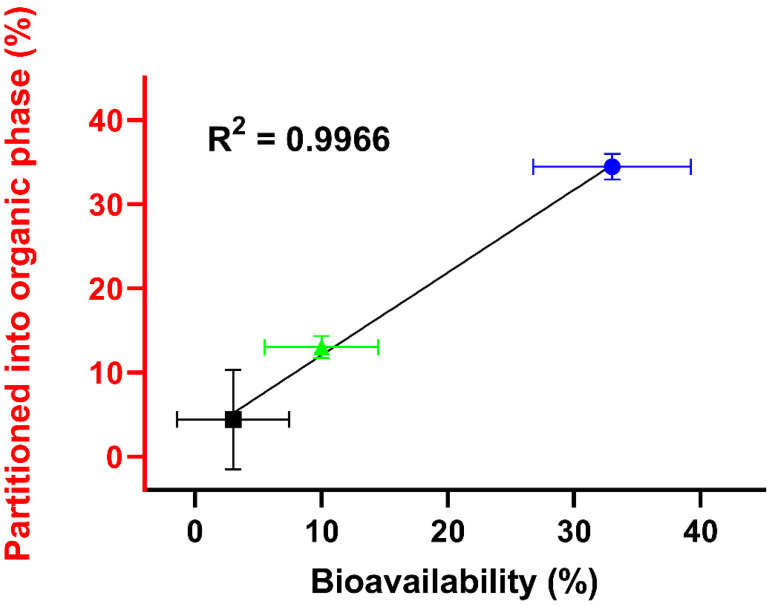
Correlation of in vitro fraction partitioned into the organic phase after 80 min, determined by biphasic dissolution and in vivo BA of neat CorA (■), CorA-povidone (●) and CorA-copovidone (▲) administered as an aqueous suspension.

In terms of relationship, a “Level C” in vitro–in vivo correlation (IVIVC) of the in vitro biphasic dissolution and the in vivo PK study was conducted (Figure 5) [36]. Therefore, the end point concentrations of the organic phase after 80 min were correlated with the BA of neat CorA and CorA-ASD formulations. The resulting correlation coefficient was 0.9966.

### 3.4. Cell Culture Experiments

#### 3.4.1. Active Transport of CorA via P-gp and BCRP

**Figure 6:**
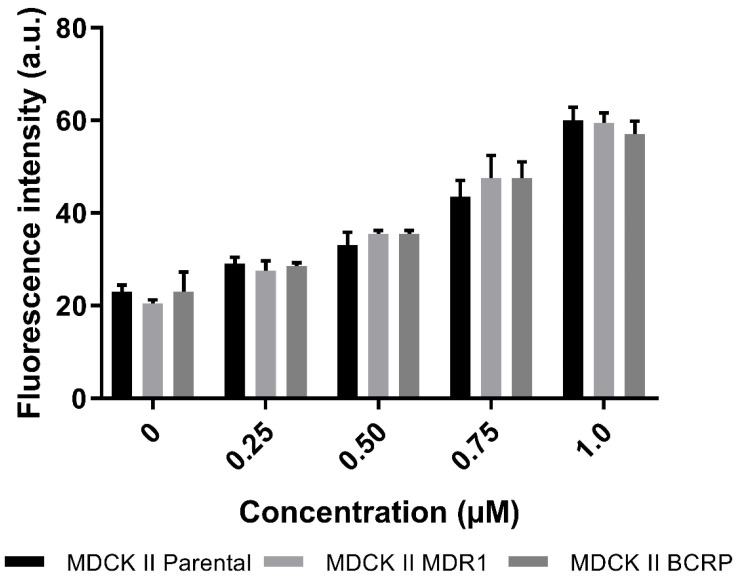
Fluorescence intensities (arbitrary unit; a.u.) of the cells in the presence of different CorA concentrations. The black bars represent the parental cells (MDCK II Parental), the light grey bars represent the cells with an overexpression of P-gp (MDCK II MDR1) and the dark grey bars represent the cells with an overexpression of BCRP (MDCK II BCRP) (mean ± SD, *n* = 3).

For substrates of P-gp and BCRP, respectively, the ratio of accumulation of the compound is at least over two-times higher in the parental cells, in comparison with the cells overexpressing these efflux transporters (Figure 6). In case of CorA there was no difference between the accumulated CorA in the different cells. Thus, there were no signs of active transport of CorA via P-gp and BCRP.

#### 3.4.2. Investigation of the Interaction with Hoechst 33342

**Figure 7:**
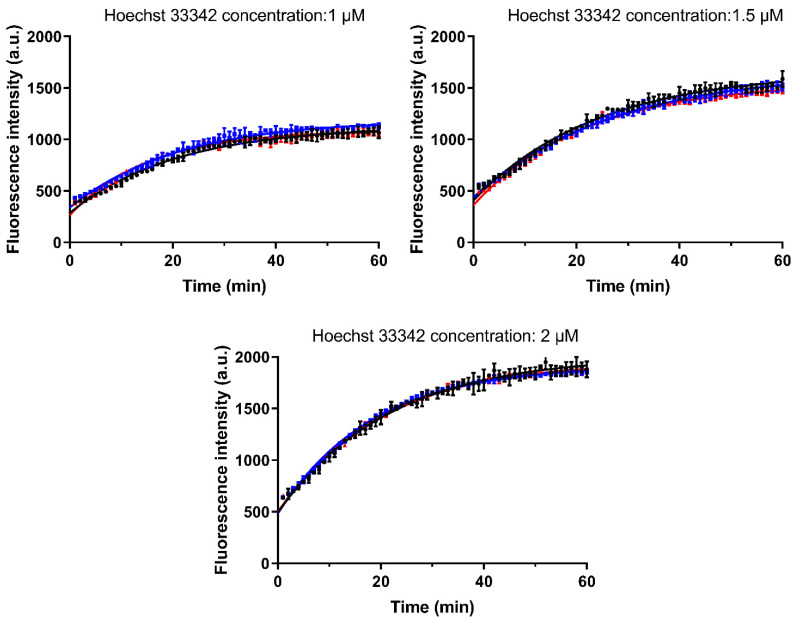
Accumulation kinetics of Hoechst 33342 alone and in the presence of CorA. Three different concentrations of Hoechst 33342 were incubated alone or together with CorA at different concentrations. (●) no CorA, (■) CorA concentration of 3.16 µM and (▲) CorA concentration of 10 µM (mean ± SD, *n* = 3). Lines were fitted using the one-phase association fit.

The accumulation of the P-gp substrate Hoechst 33342 (1.0, 1.5 and 2.0 µM) was determined in the absence and presence of CorA (3.16 µM and 10 µM) (Figure 7). For all investigated concentrations of Hoechst 33342, the presence of CorA did not affect the kinetics of uptake. The K value (representing the rate constant) as well as span (Plateau–Y0) showed no changes in the samples without and with CorA. These results confirmed that there is no interaction between CorA and the H-binding site (a suspected P-gp substrate binding site on the transporter).

### 3.5. PBPK Modeling

**Figure 8:**
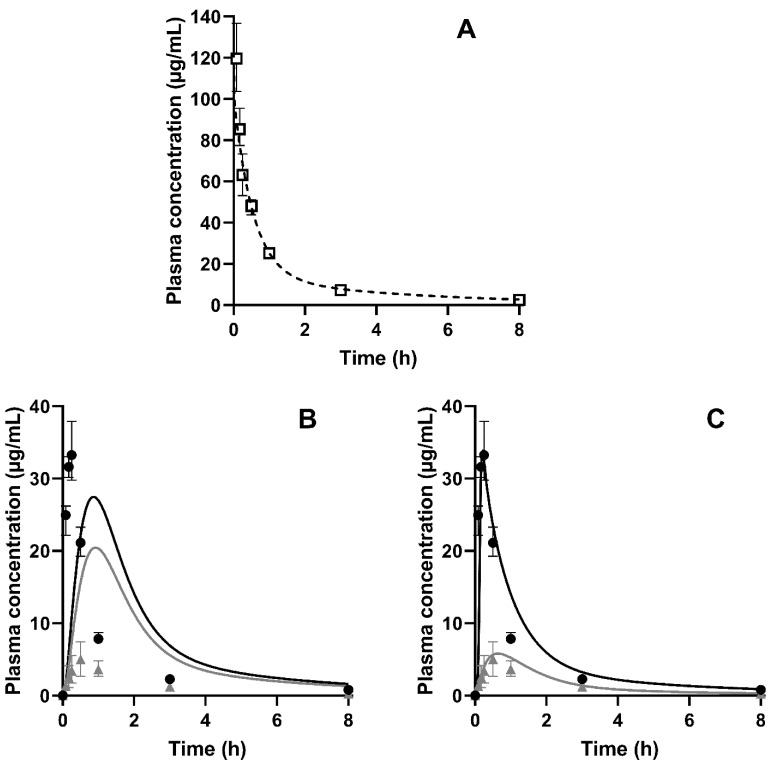
Observed and predicted plasma profiles of CorA in BALB/c mice (*n* = 4 per group); (**A**): Dashed line represents the prediction of IV administration (**B**): Lines represent the prediction of PO administration of CorA-povidone (black) and CorA-copovidone (grey), based on immediate release; (**C**): Lines represent the prediction of PO administration of CorA-povidone (black) and CorA-copovidone (grey), based on dissolution data; (□) represents observed data following the IV administration, (●) represents observed data following PO administration of CorA-povidone and (▲) represents observed data following PO administration of CorA-copovidone, respectively.

The IV data, obtained from previous experiments [14], were used to provide elimination and distribution behavior and were modelled to best fit the observed data. Measured and predicted physicochemical and physiological properties were used to build a PBPK model for the oral administration of CorA-ASD formulations. In a first step, biorelevant solubilities that were experimentally determined (Section 3.1) were incorporated into the model. Figure 8A shows the results of the PBPK modeling for the IV administration of CorA and Figure 8B shows the PO administration of CorA-ASD formulations based on in vitro-measured solubility, but without the incorporation of in vitro dissolution profiles. However, this model was not able to predict the plasma concentrations correctly. For CorA-povidone, C_max_ (27.43 µg/mL; PE: 21.18%) was underpredicted, whereas AUC_0–inf_ (64.64 µg·h/mL; PE: −54.51%) was overpredicted. For CorA-copovidone, C_max_ (20.43 µg/mL; PE: −308.60%) and AUC_0–inf_ (56.60 µg·h/mL; PE: −323.97) were overpredicted. By incorporating the dissolution profiles of the monophasic dissolution (Section 3.2.1), improved predictions were achieved (Figure 8C). For CorA-povidone, prediction errors (PEs) were <15% (C_max_: 1.71%; AUC_0–inf_: −11.31%) (Table 6). For CorA-copovidone, C_max_ and AUC_0–inf_ were slightly overpredicted (C_max_: −16.20%; AUC_0–inf_: −12.43%), which were in an acceptable range.

## 4. Discussion

Providing oral formulations which enable sufficient BA is a great challenge for novel antibiotics [37,38]. Additionally, information obtained by PK studies conducted in preclinical species are limited in terms of correlation to the future PK profile in humans due to different physiological characteristics [25,39,40]. Nevertheless, because the need for only small amounts of drug substance and being a pharmacodynamic model, mice provided a valuable PK model for CorA. In the present study the anti-infective compound CorA was evaluated using mini-scale mouse-tailored in vitro and in silico tools. As a pH-dependent soluble API, combined with sufficient permeability, CorA had poor solubility at lower pH, indicating that absorption was limited to the intestinal solubility [21]. The experimentally determined solubilities of CorA and CorA-ASD in species-specific biorelevant media allowed a comparison between the solubilities in human and mouse intestines. No precipitation occurred in both, human- and mouse-specific media and the concentration values remained at high, supersaturated levels revealing potentially high levels of dissolved drug during the small intestine transit [41]. Nevertheless, the results of the shake flask solubility setup clearly demonstrated the pH- and species-dependent differences in the solubility of CorA and CorA-ASD formulations, indicating approx. two-fold higher solubility in humans compared with mouse intestines, implying a greater absorption in humans.

Dissolution as a rate-limiting step plays an important role for drug absorption [42]. In vivo relevant monophasic and biphasic dissolution tests were conducted under biorelevant conditions to predict the in vivo dissolution performance of CorA and CorA-ASD formulations in mice. First, the monophasic setup was chosen to determine the input parameters for PBPK modeling [43]. Second, the approach of the biphasic setup was used to predict in vivo absorption, which was then correlated with the in vivo BA. The physiologic conditions, gastrointestinal pH and transit time likely influence the solubility and extent of supersaturation of APIs, and consequently, the absorption [41]. Accordingly, in vivo PK evaluation of acidic APIs such as CorA in mice could likely underestimate the fraction that is absorbed in the human intestine [44]. Likewise, gastrointestinal transit times vary between species and, consequently, the absorption window, the time and site of absorption at which dissolved API can be absorbed also varies [39,45]. This difference was taken into account by adapting the in vitro dissolution duration to the transit times of mice. The measured amount of API in the organic phase after 80 min represents the in vitro measured fraction absorbed and were used as a BA prediction [32]. Although in shake flask experiments for both formulations high supersaturated concentrations were identified, differences in dissolution performance were observed. According to the higher amount of dissolved API in the aqueous phase for the CorA-povidone formulation, more CorA partitioned into the organic phase. Similarly, the lower concentration of dissolved API with CorA-copovidone resulted in three-fold lower partitioning into the organic phase compared with CorA-povidone, which was in line with the in vivo measured differences of BA.

The PK studies in BALB/c mice further demonstrated the formulation dependent variability of BA and C_max_. Both ASD-formulations improved the performance of neat CorA and confirmed the in vitro determined superiority of CorA-povidone vs. CorA-copovidone. A good correlation of in vitro partitioned drug and in vivo BA was obtained, demonstrating the predictability of CorA and CorA formulations of the mouse-specific biphasic dissolution. Cell experiments indicated that absorption was not limited by efflux, as no interactions between CorA and P-gp or BCRP were detected. Furthermore, the accumulation kinetics of the P-gp substrate Hoechst 33342 were not influenced by CorA, confirming the first outcome and excluding CorA as an inhibitory compound at the H-binding site of P-gp. Accordingly, the CorA absorption was not influenced by efflux processes in the gut, reaffirming the prediction of the biphasic dissolution [46].

PBPK was used as a tool for the prediction of plasma concentration profiles for the CorA-ASD formulations to elucidate the in vivo relevance of the in vitro values determined in this work, since the quality of the prediction model is highly dependent on the input parameters [47,48]. The PBPK model for oral administration, based on physicochemical parameters such as pKa, logP and biorelevant solubility, was built up using IV administration data and revealed an insufficient prediction emphasized by high PEs. By incorporating the measured mouse-specific dissolution parameters, the adapted PBPK model was able to predict the plasma concentrations of CorA-ASD formulations. PE ≤ 16% in mice was considered to be successful, as predictions in mice are challenging due to difficult oral administration and limited information about mouse physiology. These results deduced that dissolution was the decisive factor for CorA formulation in mice, which was identified as the rate-limiting step for absorption, even though CorA-ASD formulations showed no solubility issues. Thus, the relevance of species-specific in vitro tools was reaffirmed and provided a more comprehensive understanding of the absorption of CorA and CorA-ASD formulations. However, similar evaluations of CorA formulations in other species, such as rats and dogs, will be necessary to provide a sound PBPK model for predicting PK performances in humans [18,49].

## 5. Conclusions

The mini-scale bi-phasic dissolution test BiPHa+ adjusted to gastric pH levels of mice resulted in level C IVIVR. Moreover, the determined dissolution data in biorelevant media enabled dissolution as the decisive mechanism governing the oral bioavailability of CorA and CorA formulations to be deduced. Hence, the presented setup confirmed the in vivo relevance for mice, thus providing a material-saving method to optimize the oral treatment of mice and select promising formulation principles even in early preclinical studies. The incorporation of solubility and dissolution into the PBPK model allowed the prediction of plasma concentration in mice, supporting the biorelevance of the mouse-specific in vitro tools. Additionally, the method can potentially be used to forecast the performance of CorA in other species via changed pH settings of the BiPHa + assay combined with PBPK-modelling to identify a later-dose regimen for humans.

## Figures and Tables

**Table 1:** Composition and physicochemical characteristics of biorelevant media simulating the small intestine environment in human and mouse in fasted and fed states.

	FaSSIF-V2- Human	FeSSIF-V2- Human	FaSSIF-V2- Mouse	FeSSIF-V2- Mouse
Lecithin (mM)	3.0	10	3.0	10
Sodium taurocholate (mM)	0.2	2	0.2	2
Glyceryl monooleate (mM)		5		5
Sodium monooleate (mM)		0.8		0.8
Sodium chloride (mM)	68.6	125.5	68.6	125.5
Sodium hydroxide (mM)	34.8	102.4	34.8	102.4
Maleic acid (mM)	19.1	71.9	19.1	71.9
Sodium hydroxide 1 N	q.s.; pH 6.5	q.s.; pH 5.8		
Hydrochloric acid 1 N			q.s.; pH 5.2	q.s.; pH 4.8
Buffer capacity (mM/∆pH)	10	25	10	25
Osmolality (mOsm/kg)	180	390	180	390
pH	6.5	5.8	5.2	4.8

**Table 2:** Composition and physicochemical characteristics of biorelevant FeSSIF-V2-like media for dissolution experiments simulating the small intestine environment in a mouse in a fed state.

Reagents	FeSSIF-V2-like
Lecithin (mM)	2
Sodium taurocholate (mM)	10
Glyceryl monooleate (mM)	5
Sodium monooleate (mM)	0.8
Sodium chloride (mM)	25.0
Potassium citrate (mM)	10
Potassium phosphate (mM)	4.3
Sodium hydroxide (mM)	10
Physicochemical characteristics	
Buffer capacity (mM/∆pH)	25.2
Osmolality (mOsm/kg)	389
pH	4.8

**Table 3:** Solubilities of neat CorA, CorA-povidone and CorA-copovidone ASD formulations over 4 h and 1 h, respectively. A: FaSSIF-V2 medium, pH 6.5; B: FeSSIF-V2, pH 5.8; C: FaSSIF-V2-mouse medium pH 5.2. D: FeSSIF-V2-mouse medium, pH 4.8. *n* = 3 (mean ± SD).

**(A) FaSSIF-V2 pH 6.5 (Human)**	**Solubility after 2 min (mg/mL)**	**Solubility after 4 h (mg/mL)**
Neat CorA	0.00 (±0.00)	0.25 (±0.06)
CorA-povidone	1.09 (±0.18)	1.24 (±0.04)
CorA-copovidone	0.96 (±0.28)	1.40 (±0.14)
**(B) FeSSIF-V2 pH 5.8 (Human)**	**Solubility after 2 min (mg/mL)**	**Solubility after 4 h (mg/mL)**
Neat CorA	0.00 (±0.00)	0.26 (±0.01)
CorA-povidone	1.06 (±0.02)	0.94 (±0.07)
CorA-copovidone	0.67 (±0.09)	0.93 (±0.05)
**(C) FaSSIF-V2-Mouse pH 5.2**	**Solubility after 2 min (mg/mL)**	**Solubility after 1 h (mg/mL)**
Neat CorA	0.00 (±0.00)	0.01 (±0.01)
CorA-povidone	0.12 (±0.01)	0.07 (±0.01)
CorA-copovidone	0.09 (±0.01)	0.19 (±0.01)
**(D) FeSSIF-V2-Mouse pH 4.8**	**Solubility after 2 min (mg/mL)**	**Solubility after 1 h (mg/mL)**
Neat CorA	0.00 (±0.00)	0.02 (±0.01)
CorA-povidone	0.66 (±0.05)	0.54 (±0.01)
CorA-copovidone	0.45 (±0.09)	0.44 (±0.08)

**Table 4:** Weibull parameters of CorA-povidone and CorA-copovidone, determined using the biorelevant monophasic dissolution setup.

Weibull Parameter	CorA-Povidone	CorA-Copovidone
Maximum of released API (%)	65.43	16.78
Time lag (h)	0	0
Shape factor	3.73	0.85
Time scale (hb)	2 × 10−4	0.47
R2	0.92	0.92

**Table 5:** Pharmacokinetic parameters of CorA and CorA formulations after IV administration of a CorA-solution (36 mg/kg) and PO administration of the solid CorA-ASD formulations CorA-povidone and CorA copovidone (36 mg/kg) in female BALB/c mice (median ± IQR, *n* = 4 per group).

	AUC_0–inf_ (µg·h/mL)	C_max_ (µg/mL)	Tmax (min)	BA (%)
Reference of CorA–IV ^#^	127.7 (110.2–149.0)	119.6 (103.7–136.7)	5 *	100 **
Neat CorA	4.5 (3.8–7.4)	0.9 (0.6–1.1)	60 (53–90)	3
CorA-povidone	41.9 (39.6–46.5)	33.2 (30.3–37.9)	15 (10–15)	33
CorA-copovidone	13.4 (11.4–17.4)	5.0 (3.0–7.4)	30 (30–38)	10

* First measured value; ** IV result median set to 100%; ^#^ [14].

**Table 6:** Observed and predicted values of C_max_ and AUC_0–inf_ of CorA-povidone and CorA-copovidone after oral administration.

**Parameters**	**CorA-Povidone**				
	**Observed**	**Predicted** **(w/o Dissolution)**	**PE (%)** **(w/o Dissolution)**	**Predicted** **(w/ Dissolution)**	**PE (%)** **(w/ Dissolution)**
C_max_ (µg/mL)	33.24	27.43	21.18	33.80	−1.68
AUC_0–inf_ (µg·h/mL)	41.83	64.63	−54.51	46.44	−11.02
**Parameters**	**CorA-Copovidone**				
	**Observed**	**Predicted** **(w/o Dissolution)**	**PE (%)** **(w/o Dissolution)**	**Predicted** **(w/ Dissolution)**	**PE (%) ** **(w/ Dissolution)**
C_max_ (µg/mL)	5.00	20.43	−308.60	5.80	−16.00
AUC_0–inf_ (µg·h/mL)	13.35	56.60	−323.97	15.00	−12.36

## Data Availability

The data presented in this study are available in the research article.

## Supplementary Materials

The following supporting information can be downloaded at: https://www.mdpi.com/article/10.3390/pharmaceutics14081657/s1, Figure S1: X-ray powder diffraction (XRPD) diffractograms of neat CorA, CorA-povidone and CorA-copovidone. XRPD studies were performed in transmission mode on a X’Pert MRD Pro (PANalytical, Almelo, The Netherlands). Nickel filtered CuKα1 radiation was generated at 45 kV and 40 mA and the scans were performed from 8° to 45° 2θ with a step size of 0.017° 2θ; Table S1: Composition of buffer and surfactant concentrate for simulating the environment in the small intestine in a fed state; Table S2: Physicochemical, biopharmaceutical and physiological properties to perform PBPK simulations; Table S3: Properties of neat CorA and spray dried CorA-ASD formulations.

## Abbreviations

CorA: corallopyronin A; API, active pharmaceutical ingredient; BA, bioavailability; ASD, amorphous solid dispersion; PO, per os; PBPK, physiologically based pharmacokinetic; HPLC, high-performance liquid chromatography; ANOVA, analysis of variance; AUC, area under the curve; C_max_, maximum plasma concentration; Tmax, time at which maximum plasma concentration is observed; IV, intravenous; PK, pharmacokinetic; P-gp, P-glycoprotein; BCRP, breast cancer resistance protein; DMEM, Dulbecco’s Modified Eagle’s Medium; PBS, phosphate-buffered saline; PE, prediction error; IVIVC, in vitro–in vivo correlation.
