# Supplementary material for: In Vitro–In Vivo Relationship in Mini-Scale—Enabling Formulations of Corallopyronin A
Source: Pharmaceutics. 2022 Aug 9;14(8):1657. doi: 10.3390/pharmaceutics14081657 (PMC9414514; doi:10.3390/pharmaceutics14081657)
Supplement: Supplementary file 1 [file pharmaceutics-14-01657-s001.zip › pharmaceutics-1837520-supplementary.pdf]

Supplementary Materials: In vitro In Vivo Relationship in Mini-Scale—Enabling Formulations for Corallopyronin A

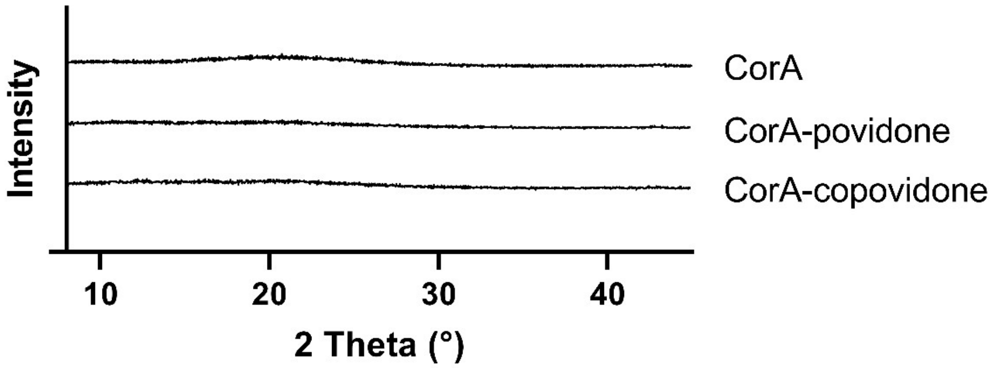

Figure S1. X-ray powder diffraction (XRPD) diffractograms of neat CorA, CorA-povidone and CorA-copovidone. XRPD studies were performed in transmission mode on a X'Pert MRD Pro (PANalytical, Almelo, The Netherlands). Nickel filtered CuKα1 radiation was generated at 45 kV and 40 mA and the scans were performed from 8 ° to 45 °2θ with a step size of 0.017 ° 2θ.

Table S1. Composition of buffer and surfactant concentrate for simulating the environment in the small intestine in a fed state.

Composition	Concentration (mM)	added V for pH 4.8 / fed state (mL)	Concentration in 50 mL (mM)
**Buffer**		0.950	
Potassium citrate	525		10.0
Potassium phosphate	225		4.3
Sodium hydroxide	527		10.0
**Surfactants**		1.000	
Lecithin	100		2
Sodium taurocholate	500		10
Glyceryl monooleate	250		5
Sodium monooleate	40		0.8

Table S2. Physicochemical, biopharmaceutical and physiological properties to perform PBPK simulations.

**Input Parameter**	Value/Selection User Defined	Value/Selection Default Settings	Reference
**Physicochemical Properties**			
Molecular weight (g/mol)	527	527	[14]
pKa	3.6	5.1	[14]
logP	5.4		[14]
**Biopharmaceutic Properties**			
Caco-2 Papp (cm/s x E10)	2.0		Experimentally given
Dose Volume (mL)	0.2		Experimentally given
pH at reference solubility	1		[14]
Solubility at reference pH (mg/mL)	0.00011		[14]
Mean Precipitation Time (sec)	900	900	Default
Biorelevant in vitro solubilities: Solubilization Ratio	2.40×10^6 (CorA-povidone) 1.31×10^6 (CorA-copovidone)	3.44×10^5 (CorA-povidone) 3.44×10^5 (CorA-copovidone)	Experimentally given
Fraction plasma unbound (%)	0.1		Experimentally given
Blood/plasma concentration ratio	0.75	0.75	Predicted by ADMET predictor
Body weight mouse (kg)	0.02	0.02	Experimentally given
Clearance (L/h)	0.006		Experimentally given
Volume of distribution (L)	0.016		Experimentally given
**ACAT™ Model Parameters:**			
Gut physiology	Mouse - Physiological - Fed	Mouse - Physiological - Fed	

Table S3. Properties of neat CorA and spray dried CorA-ASD-formulations.

**Parameter**	Neat CorA	CorA-povidone	CorA-copovidone	Reference
Glass transition temperature (°C)	5	116	84	[14]
*Solid-state	Amorphous	Amorphous	Amorphous	
**D50 (µm)	Not applicable	12.65	10.83	
**D90 (µm)	Not applicable	21.03	17.48	
**D10 (µm)	Not applicable	7.93	6.84	

*Differential scanning calorimetry (DSC) and XRPD measurements demonstrated the completely amorphous state of neat CorA and CorA-ASD-formulations. DSC measurements showed only one glass transition temperature, indicating no phase separation.

**Particle size distributions were measured with a Horiba LA-960 laser diffractometer (Horiba, Kyoto, Japan). A red laser diode with 650 nm wavelength (5 mW) and a blue light emitting diode (3 mW) with 405 nm wavelength were used as light sources. Samples were measured by the wet dispersion method in n-hexane with 0.1% Span 80 (v/v). A magnetic stirrer was placed in the cuvette to agitate the sample.
